# The genome sequence of the large yellow underwing, *Noctua pronuba *(Linnaeus, 1758)

**DOI:** 10.12688/wellcomeopenres.17747.1

**Published:** 2022-04-01

**Authors:** Douglas Boyes, Peter W.H. Holland, University of Oxford and Wytham Woods Genome Acquisition Lab, Darwin Tree of Life Barcoding collective, Wellcome Sanger Institute Tree of Life programme, Wellcome Sanger Institute Scientific Operations: DNA Pipelines collective, Tree of Life Core Informatics collective, Darwin Tree of Life Consortium

**Affiliations:** 1 UK Centre for Ecology and Hydrology, Wallingford, Oxfordshire, UK; 2 Department of Zoology, University of Oxford, Oxford, UK

**Keywords:** Noctua pronuba, large yellow underwing, genome sequence, chromosomal, Lepidoptera

## Abstract

We present a genome assembly from an individual female *Noctua pronuba *(the large yellow underwing; Arthropoda; Insecta; Lepidoptera; Noctuidae). The genome sequence is 529 megabases in span. The complete assembly is scaffolded into 32 chromosomal pseudomolecules, with the W and Z sex chromosome assembled. The mitochondrial genome was also assembled and is 15.3 kilobases in length.

## Species taxonomy

Eukaryota; Metazoa; Ecdysozoa; Arthropoda; Hexapoda; Insecta; Pterygota; Neoptera; Endopterygota; Lepidoptera; Glossata; Ditrysia; Noctuoidea; Noctuidae; Noctuinae; Noctuini; Noctua; *Noctua fimbriata* (Linnaeus, 1758) (NCBI:txid214277).

## Background

*Noctua pronuba* (large yellow underwing) is a widespread noctuid moth found across Eurasia and North Africa, and one of the most familiar moths in the UK with hundreds of adults commonly caught in a single light trap. The larvae are polyphagous, feeding on a wide range of grasses and herbaceous plants, and have bright green colouration in early instars, changing to brown in later instars. The moth was recorded in Canada in 1979 and has spread south since its accidental introduction; *N. pronuba* is now an occasional pest of commercial crops in the United States, where the larvae are known as winter cutworm ( Difonzo & Russell, 2010). In a major outbreak in Michigan in 2007, vast numbers of larvae caused defoliation of fields of alfalfa, rye, oat and winter wheat, and reached public nuisance proportions ( Difonzo & Russell, 2010).

The forewings of the adult moth may be light brown, ochreous or dark-purplish brown, with colour controlled genetically through polymorphism at unknown loci modified by sex-linked genes ( Cook & Sarsam, 1981). The hindwings, which are completely hidden at rest, are bright orange-yellow with a black band. When disturbed and the moth takes flight, the yellow hindwings are suddenly revealed, plausibly acting as ‘flash colouration’ to startle predators. In Europe, *N. pronuba* is a strongly migratory species: vertical-looking entomological radars sited in the UK have detected large numbers of individuals flying north in spring or south in autumn ( Chapman *et al.*, 2010). Indeed, in experiments using moths tethered to a flight mill *N. pronuba* was found to be one of the most mobile noctuid species as measured by both maximum flight speed and total distance covered in one night ( Jones *et al.*, 2015). Migration direction is thought to be affected by use of magnetic compass sense, coupled with the ability to detect and utilise favourably-directed winds ( Baker & Mather, 1982; Reynolds *et al.*, 2010).

The genome of *N. pronuba* was sequenced as part of the Darwin Tree of Life Project, a collaborative effort to sequence all of the named eukaryotic species in the Atlantic Archipelago of Britain and Ireland. Here we present a chromosomally complete genome sequence for *N. pronuba*, based on one female specimen from Wytham Woods, Oxfordshire, UK.

## Genome sequence report

**Figure 1.**
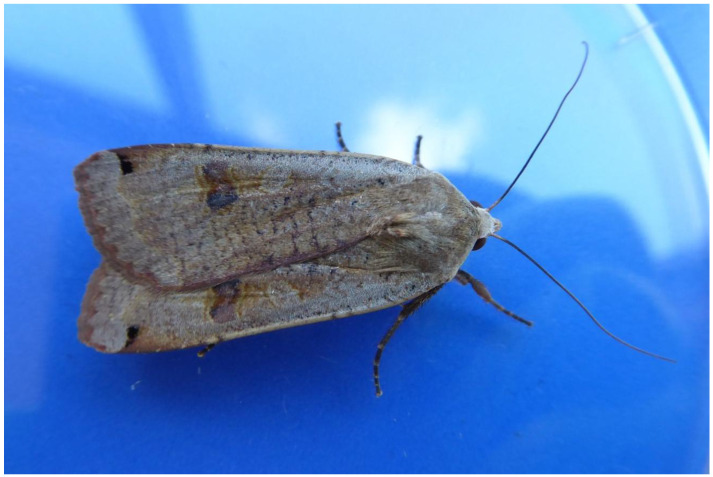
Image of the ilNocPron1 specimen taken during preservation and processing.

The genome was sequenced from a single female *N. pronuba* collected from Wytham Woods, Oxfordshire, UK (latitude 51.772, longitude -1.338) ( Figure 1). A total of 47-fold coverage in Pacific Biosciences single-molecule HiFi long reads and 71-fold coverage in 10X Genomics read clouds were generated. Primary assembly contigs were scaffolded with chromosome conformation Hi-C data. Manual assembly curation corrected 21 missing/misjoins and removed 6 haplotypic duplications, reducing the assembly length by 0.02% and the scaffold number by 25.76%, and increasing the scaffold N50 by 1.57%.

**Figure 2.**
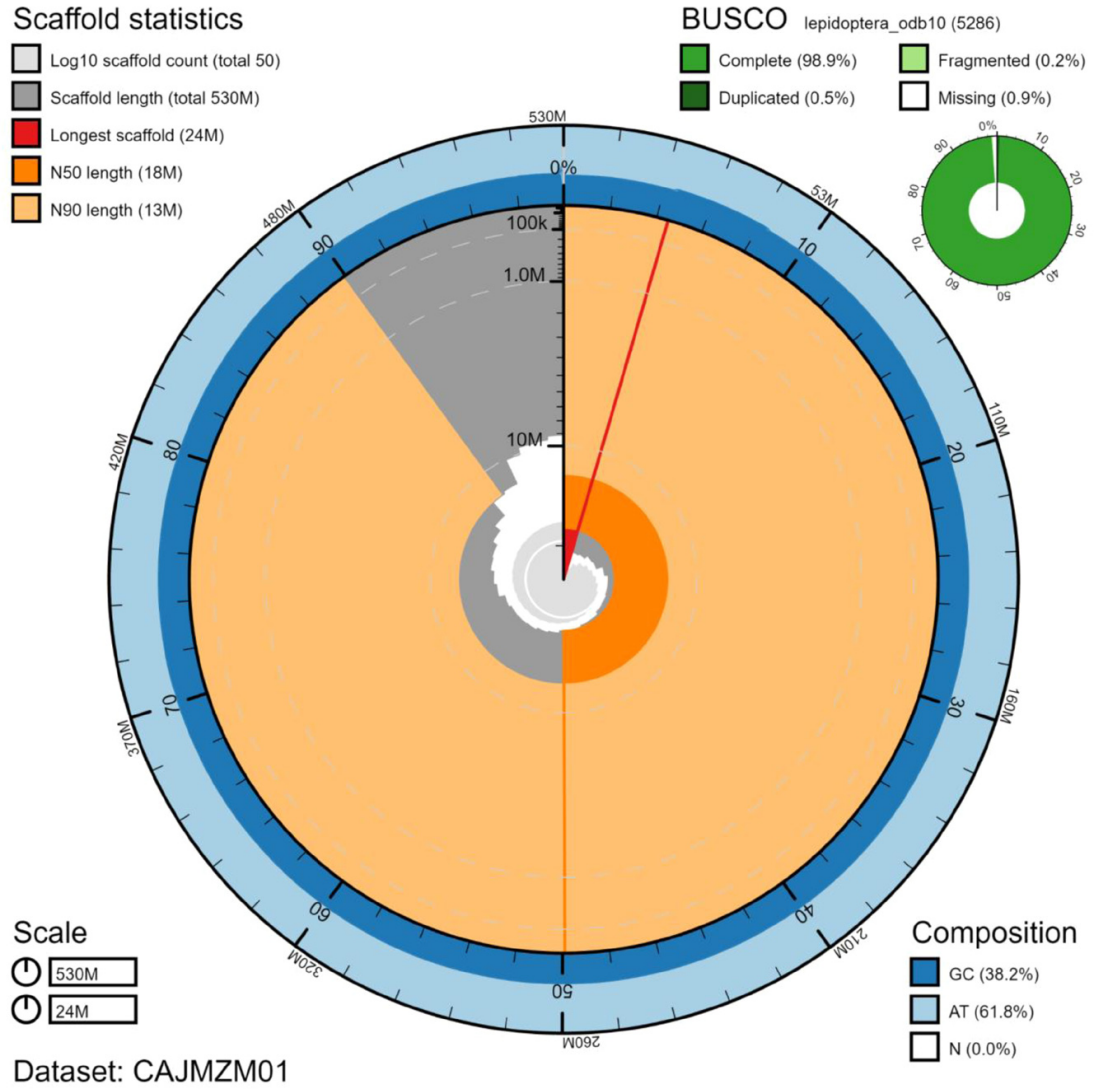
Genome assembly of *Noctua pronuba*, ilNocPron1.1: metrics. The BlobToolKit Snailplot shows N50 metrics and BUSCO gene completeness. The main plot is divided into 1,000 size-ordered bins around the circumference with each bin representing 0.1% of the 529,194,529 bp assembly. The distribution of chromosome lengths is shown in dark grey with the plot radius scaled to the longest chromosome present in the assembly (24,140,489 bp, shown in red). Orange and pale-orange arcs show the N50 and N90 chromosome lengths (17,891,575 and 12,542,342 bp), respectively. The pale grey spiral shows the cumulative chromosome count on a log scale with white scale lines showing successive orders of magnitude. The blue and pale-blue area around the outside of the plot shows the distribution of GC, AT and N percentages in the same bins as the inner plot. A summary of complete, fragmented, duplicated and missing BUSCO genes in the lepidoptera_odb10 set is shown in the top right. An interactive version of this figure is available at https://blobtoolkit.genomehubs.org/view/ilNocPron1.1/dataset/CAJMZM01/snail.

The final assembly has a total length of 529 Mb in 49 sequence scaffolds with a scaffold N50 of 17.9 Mb ( Table 1). Of the assembly sequence, 99.89% was assigned to 32 chromosomal-level scaffolds, representing 30 autosomes (numbered by sequence length), and the W and Z sex chromosome ( Figure 2– Figure 5; Table 2). The assembly has a BUSCO ( Manni *et al.*, 2021) completeness of 98.9% using the lepidoptera_odb10 reference set. While not fully phased, the assembly deposited is of one haplotype. Contigs corresponding to the second haplotype have also been deposited.

**Table 1.** Genome data for *Noctua pronuba*, ilNocPron1.1.

***Project accession data***	
Assembly identifier	ilNocPron1.1
Species	*Noctua pronuba*
Specimen	ilNocPron1
NCBI taxonomy ID	NCBI:txid214277
BioProject	PRJEB43815
BioSample ID	SAMEA7519837
Isolate information	Female, whole organism
***Raw data accessions***	
PacificBiosciences SEQUEL II	ERR6590587
10X Genomics Illumina	ERR6054676-ERR6054679
Hi-C Illumina	ERR6054680-ERR6054682
***Genome assembly***	
Assembly accession	GCA_905163415.1
*Accession of alternate haplotype*	GCA_905220345.1
Span (Mb)	529
Number of contigs	74
Contig N50 length (Mb)	16.2
Number of scaffolds	49
Scaffold N50 length (Mb)	17.9
Longest scaffold (Mb)	21.7
BUSCO * genome score	C:98.9%[S:98.3%,D:0.5%], F:0.2%,M:0.9%,n:5286

*BUSCO scores based on the lepidoptera_odb10 BUSCO set using v5.1.2. C= complete [S= single copy, D=duplicated], F=fragmented, M=missing, n=number of orthologues in comparison. A full set of BUSCO scores is available at https://blobtoolkit.genomehubs.org/view/ilNocPron1.1/dataset/CAJMZM01/busco

**Figure 3.**
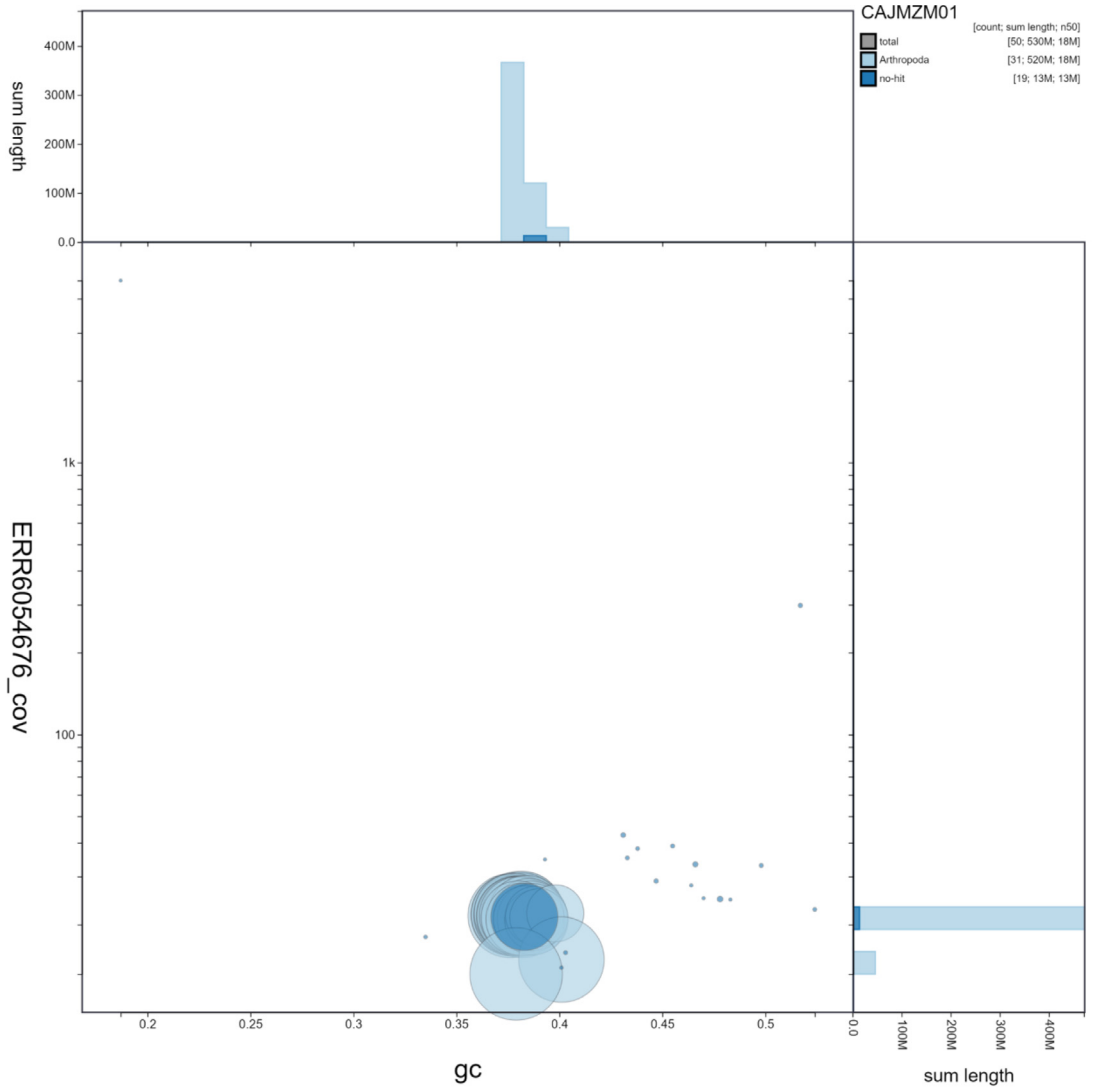
Genome assembly of *Noctua pronuba*, ilNocPron1.1: GC coverage. BlobToolKit GC-coverage plot. Scaffolds are coloured by phylum. Circles are sized in proportion to scaffold length. Histograms show the distribution of scaffold length sum along each axis. An interactive version of this figure is available at https://blobtoolkit.genomehubs.org/view/ilNocPron1.1/dataset/CAJMZM01/blob.

**Figure 4.**
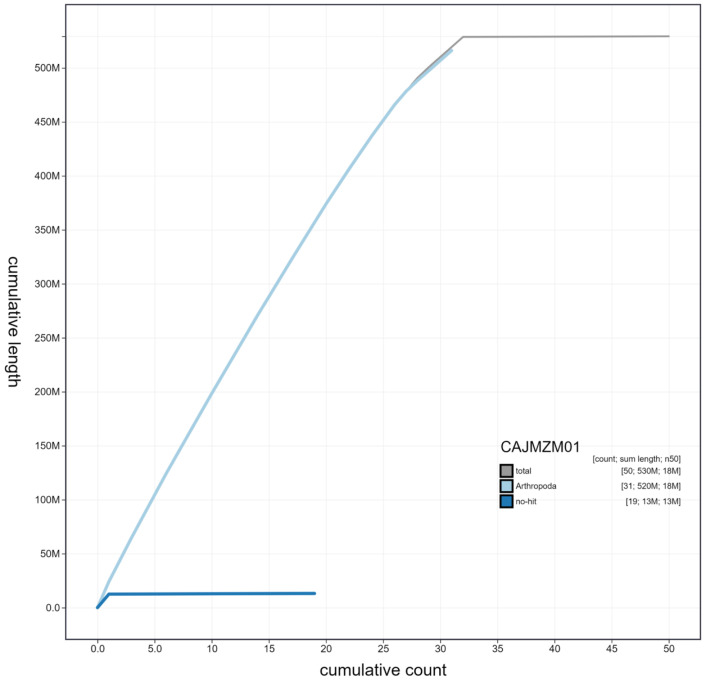
Genome assembly of *Noctua pronuba*, ilNocPron1.1: cumulative sequence. BlobToolKit cumulative sequence plot. The grey line shows cumulative length for all scaffolds. Coloured lines show cumulative lengths of scaffolds assigned to each phylum using the buscogenes taxrule. An interactive version of this figure is available at https://blobtoolkit.genomehubs.org/view/ilNocPron1.1/dataset/CAJMZM01/cumulative.

**Figure 5.**
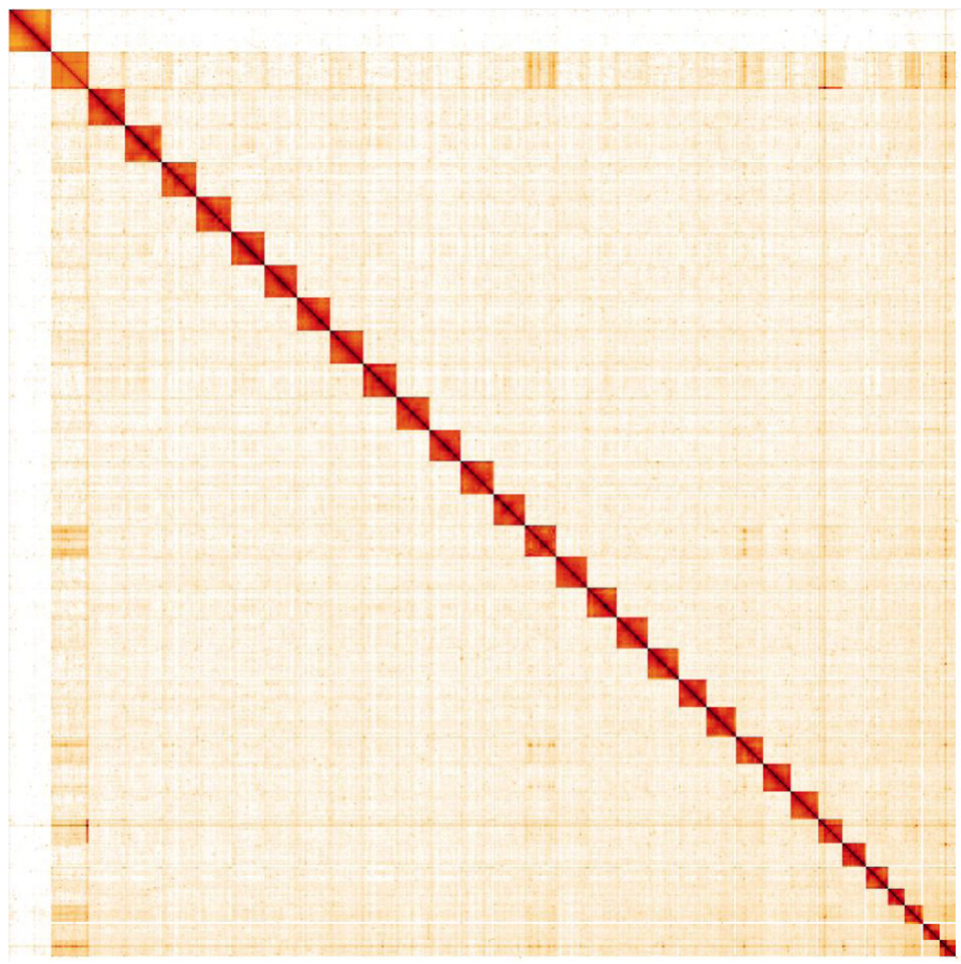
Genome assembly of *Noctua pronuba*, ilNocPron1.1: Hi-C contact map. Hi-C contact map of the ilNocPron1.1 assembly, visualised in HiGlass. Chromosomes are shown in order of size from left to right and top to bottom.

**Table 2.** Chromosomal pseudomolecules in the genome assembly of *Noctua pronuba*, ilNocPron1.1.

INSDC accession	Chromosome	Size (Mb)	GC%
LR999893.1	1	20.39	38.0
LR999894.1	2	19.95	38.2
LR999895.1	3	19.85	37.6
LR999896.1	4	19.06	37.7
LR999897.1	5	18.68	38.2
LR999898.1	6	18.65	37.8
LR999899.1	7	18.55	37.7
LR999900.1	8	18.48	38.1
LR999901.1	9	18.20	38.2
LR999902.1	10	18.14	37.8
LR999903.1	11	17.91	37.8
LR999904.1	12	17.89	37.9
LR999905.1	13	17.62	38.0
LR999906.1	14	17.46	38.2
LR999907.1	15	17.25	37.9
LR999908.1	16	17.15	38.1
LR999909.1	17	17.08	38.3
LR999910.1	18	16.84	38.0
LR999911.1	19	16.20	38.4
LR999912.1	20	15.88	38.5
LR999913.1	21	15.49	38.6
LR999914.1	22	15.37	38.0
LR999915.1	23	14.84	38.2
LR999916.1	24	14.16	38.7
LR999917.1	25	12.54	38.3
LR999918.1	26	12.31	38.4
LR999919.1	27	10.04	38.8
LR999920.1	28	9.48	38.8
LR999921.1	29	9.39	39.0
LR999922.1	30	9.09	39.8
LR999892.1	W	20.55	40.1
LR999891.1	Z	24.14	37.8
LR999923.1	MT	0.02	18.9
-	Unplaced	0.55	45.4

## Methods

### Sample acquisition, DNA extraction and sequencing

A single female *N. pronuba* (ilNocPron1) was collected from Wytham Woods, Oxfordshire, UK (latitude 51.772, longitude -1.338) by Douglas Boyes, UKCEH, and identified by the same individual. The specimen was collected using a light trap in woodland, preserved on dry ice prior to transfer to the Wellcome Sanger Institute.

DNA was extracted from whole organism tissue at the Wellcome Sanger Institute (WSI) Scientific Operations core from the whole organism using the Qiagen MagAttract HMW DNA kit, according to the manufacturer’s instructions. Pacific Biosciences HiFi circular consensus and 10X Genomics read cloud sequencing libraries were constructed according to the manufacturers’ instructions. Sequencing was performed by the Scientific Operations core at the Wellcome Sanger Institute on Pacific Biosciences SEQUEL II and Illumina HiSeq X instruments. Hi-C data were generated from remaining whole organism tissue using the Arima Hi-C v2 kit and sequenced on HiSeq X.

### Genome assembly

Assembly was carried out with HiCanu ( Nurk *et al.*, 2020); haplotypic duplication was identified and removed with purge_dups ( Guan *et al.*, 2020). One round of polishing was performed by aligning 10X Genomics read data to the assembly with longranger align, calling variants with freebayes ( Garrison & Marth, 2012). The assembly was then scaffolded with Hi-C data ( Rao *et al.*, 2014) using SALSA2 ( Ghurye *et al.*, 2019). The assembly was checked for contamination and corrected using the gEVAL system ( Chow *et al.*, 2016) as described previously ( Howe *et al.*, 2021). Manual curation ( Howe *et al.*, 2021) was performed using gEVAL, HiGlass ( Kerpedjiev *et al.*, 2018) and Pretext. The mitochondrial genome was assembled using MitoHiFi ( Uliano-Silva *et al.*, 2021), which performed annotation using MitoFinder ( Allio *et al.*, 2020). The genome was analysed and BUSCO scores generated within the BlobToolKit environment ( Challis *et al.*, 2020). Table 3 contains a list of all software tool versions used, where appropriate.

**Table 3.** Software tools used.

Software tool	Version	Source
HiCanu	2.1	Nurk *et al.*, 2020
purge_dups	1.2.3	Guan *et al.*, 2020
SALSA2	2.2	Ghurye *et al.*, 2019
longranger align	2.2.2	https://support.10xgenomics.com/genome-exome/ software/pipelines/latest/advanced/other-pipelines
freebayes	1.3.1-17-gaa2ace8	Garrison & Marth, 2012
MitoHiFi	1	Uliano-Silva *et al.*, 2021
gEVAL	N/A	Chow *et al.*, 2016
PretextView	0.1.x	https://github.com/wtsi-hpag/PretextView
HiGlass	1.11.6	Kerpedjiev *et al.*, 2018
BlobToolKit	2.6.4	Challis *et al.*, 2020

### Ethics/compliance issues

The materials that have contributed to this genome note have been supplied by a Darwin Tree of Life Partner. The submission of materials by a Darwin Tree of Life Partner is subject to the Darwin Tree of Life Project Sampling Code of Practice. By agreeing with and signing up to the Sampling Code of Practice, the Darwin Tree of Life Partner agrees they will meet the legal and ethical requirements and standards set out within this document in respect of all samples acquired for, and supplied to, the Darwin Tree of Life Project. Each transfer of samples is further undertaken according to a Research Collaboration Agreement or Material Transfer Agreement entered into by the Darwin Tree of Life Partner, Genome Research Limited (operating as the Wellcome Sanger Institute), and in some circumstances other Darwin Tree of Life collaborators.

## Data availability

European Nucleotide Archive: Noctua pronuba (large yellow underwing). Accession number PRJEB43815; https://identifiers.org/ena.embl/PRJEB43815.

The genome sequence is released openly for reuse. The *N. pronuba* genome sequencing initiative is part of the Darwin Tree of Life (DToL) project. All raw sequence data and the assembly have been deposited in INSDC databases. The genome will be annotated and presented through the Ensembl pipeline at the European Bioinformatics Institute. Raw data and assembly accession identifiers are reported in Table 1.

## Author information

Members of the University of Oxford and Wytham Woods Genome Acquisition Lab are listed here: https://doi.org/10.5281/zenodo.5746938.

Members of the Darwin Tree of Life Barcoding collective are listed here: https://doi.org/10.5281/zenodo.5744972.

Members of the Wellcome Sanger Institute Tree of Life programme are listed here: https://doi.org/10.5281/zenodo.6125027.

Members of Wellcome Sanger Institute Scientific Operations: DNA Pipelines collective are listed here: https://doi.org/10.5281/zenodo.5746904.

Members of the Tree of Life Core Informatics collective are listed here: https://doi.org/10.5281/zenodo.6125046.

Members of the Darwin Tree of Life Consortium are listed here: https://doi.org/10.5281/zenodo.5638618.

## References

[ref-1] Allio R Schomaker-Bastos A Romiguier J : MitoFinder: Efficient Automated Large-Scale Extraction of Mitogenomic Data in Target Enrichment Phylogenomics. *Mol Ecol Resour.* 2020;20(4):892–905. 10.1111/1755-0998.13160 32243090 PMC7497042

[ref-2] Baker RR Mather JG : Magnetic Compass Sense in the Large Yellow Underwing Moth, *Noctua Pronuba* L. *Anim Behav.* 1982;30(2):543–48. 10.1016/S0003-3472(82)80067-5

[ref-3] Challis R Richards E Rajan J : BlobToolKit - Interactive Quality Assessment of Genome Assemblies. *G3 (Bethesda).* 2020;10(4):1361–74. 10.1534/g3.119.400908 32071071 PMC7144090

[ref-18] Chapman JW Nesbit RL Burgin LE : Flight orientation behaviors promote optimal migration trajectories in high-flying insects. *Science.* 2010;327(5966):682–685. 10.1126/science.1182990 20133570

[ref-4] Chow W Brugger K Caccamo M : gEVAL - a web-based browser for evaluating genome assemblies. *Bioinformatics.* 2016;32(16):2508–10. 10.1093/bioinformatics/btw159 27153597 PMC4978925

[ref-5] Cook LM Sarsam V : Polymorphism in the Moth *Noctua Pronuba* (L.). *Heredity.* 1981;46(3):443–47. 10.1038/hdy.1981.51

[ref-6] Difonzo C Russell H : *Noctua Pronuba* (Lepidoptera: Noctuidae): An Outbreak in Emails. *J Integr Pest Manag.* 2010;1(1):B1–6. 10.1603/IPM10005

[ref-7] Garrison E Marth G : Haplotype-Based Variant Detection from Short-Read Sequencing.arXiv: 1207.3907.2012. Reference Source

[ref-8] Ghurye J Rhie A Walenz BP : Integrating Hi-C Links with Assembly Graphs for Chromosome-Scale Assembly. *PLoS Comput Biol.* 2019;15(8):e1007273. 10.1371/journal.pcbi.1007273 31433799 PMC6719893

[ref-9] Guan D McCarthy SA Wood J : Identifying and Removing Haplotypic Duplication in Primary Genome Assemblies. *Bioinformatics.* 2020;36(9):2896–98. 10.1093/bioinformatics/btaa025 31971576 PMC7203741

[ref-10] Howe K Chow W Collins J : Significantly Improving the Quality of Genome Assemblies through Curation. *GigaScience.* 2021;10(1):giaa153. 10.1093/gigascience/giaa153 33420778 PMC7794651

[ref-11] Jones HB Lim KS Bell JR : Quantifying Interspecific Variation in Dispersal Ability of Noctuid Moths Using an Advanced Tethered Flight Technique. *Ecol Evol.* 2015;6(1):181–90. 10.1002/ece3.1861 26811783 PMC4716516

[ref-12] Kerpedjiev P Abdennur N Lekschas F : HiGlass: Web-Based Visual Exploration and Analysis of Genome Interaction Maps. *Genome Biol.* 2018;19(1):125. 10.1186/s13059-018-1486-1 30143029 PMC6109259

[ref-13] Manni M Berkeley MR Seppey M : BUSCO Update: Novel and Streamlined Workflows along with Broader and Deeper Phylogenetic Coverage for Scoring of Eukaryotic, Prokaryotic, and Viral Genomes. *Mol Biol Evol.* 2021;38(10):4647–54. 10.1093/molbev/msab199 34320186 PMC8476166

[ref-14] Nurk S Walenz BP Rhie A : HiCanu: Accurate Assembly of Segmental Duplications, Satellites, and Allelic Variants from High-Fidelity Long Reads. *Genome Res.* 2020;30(9):1291–1305. 10.1101/gr.263566.120 32801147 PMC7545148

[ref-15] Rao SSP Huntley MH Durand NC : A 3D Map of the Human Genome at Kilobase Resolution Reveals Principles of Chromatin Looping. *Cell.* 2014;159(7):1665–80. 10.1016/j.cell.2014.11.021 25497547 PMC5635824

[ref-16] Reynolds AM Reynolds DR Smith AD : Orientation Cues for High-Flying Nocturnal Insect Migrants: Do Turbulence-Induced Temperature and Velocity Fluctuations Indicate the Mean Wind Flow? *PLoS One.* 2010;5(12):e15758. 10.1371/journal.pone.0015758 21209956 PMC3012097

[ref-17] Uliano-Silva M Nunes JGF Krasheninnikova K : marcelauliano/MitoHiFi: mitohifi_v2.0.2021. 10.5281/zenodo.5205678

