# Supplementary material for: The genome sequence of the large yellow underwing, Noctua pronuba (Linnaeus, 1758)
Source: Wellcome Open Res. 2022 Apr 1;7:119. [Version 1] doi: 10.12688/wellcomeopenres.17747.1 (PMC9975414; doi:10.12688/wellcomeopenres.17747.1)
Supplement: Peer review history

# Peer review: Reviewer response for version 1

**DOI:** 10.21956/wellcomeopenres.19641.r53302

**Published:** 2022-12-08

**Authors:** Joost van den Heuvel

**Affiliations:** 1 Laboratory of Genetics, Wageningen University & Research, Wageningen, The Netherlands

This data note contains the description of the chromosome level assembly of the genome of *Noctua pronuba*. Data and analysis are well suited and state of the art, resulting in a highly complete and chromosome level assembly, including two sex chromosomes and an alternate haplotype. This assembly will be useful to different fields of research as *Noctua pronuba* is an extremely common species, shows interesting phenotypes but, is also a pest species in North America since the early 80s.

Are sufficient details of methods and materials provided to allow replication by others?

Partly

Is the rationale for creating the dataset(s) clearly described?

Yes

Are the datasets clearly presented in a useable and accessible format?

Yes

Are the protocols appropriate and is the work technically sound?

Yes

Reviewer Expertise:

population genetics, entomology, evolution, life histories

I confirm that I have read this submission and believe that I have an appropriate level of expertise to confirm that it is of an acceptable scientific standard.

# Peer review: Reviewer response for version 1

**DOI:** 10.21956/wellcomeopenres.19641.r53301

**Published:** 2022-12-02

**Authors:** Boyd A. Mori

**Affiliations:** 1 Department of Agricultural, Food and Nutritional Science, University of Alberta, Edmonton, AB, Canada

This data note provides the first genome assembly for the large yellow underwing moth, *Noctua pronuba*. The species is widespread throughout the Palearctic, and the larvae of which are an occasional crop pest. 

 This is an additional species to the Tree of Life project. The assembly uses the current leading methods in genome sequencing and assembly. Methods although sparse, are clear. One potential caveat for all of these data notes would be to include a supplementary file with the parameters used for all programs in the assembly pipeline to enhance reproducibility.

 Standard metrics all indicate a high quality genome assembly.

Are sufficient details of methods and materials provided to allow replication by others?

Yes

Is the rationale for creating the dataset(s) clearly described?

Yes

Are the datasets clearly presented in a useable and accessible format?

Yes

Are the protocols appropriate and is the work technically sound?

Yes

Reviewer Expertise:

agricultural entomology, insect genomics

I confirm that I have read this submission and believe that I have an appropriate level of expertise to confirm that it is of an acceptable scientific standard.
